# ‘I Want Everyone to Have It, and Everyone to Be on It’: A Feasibility Study of the Transforming Long Covid Intervention

**DOI:** 10.1111/hex.70681

**Published:** 2026-05-03

**Authors:** Sarahjane Belton, Hannah Goss, Enda Whyte, Noel McCaffrey, Sophie Gibney, Kate Sheridan

**Affiliations:** ^1^ School of Health and Human Performance Dublin City University Dublin Ireland; ^2^ ExWell Medical Dublin Ireland

**Keywords:** autonomic dysfunction, chronic illness, persistent physical symptoms, rehabilitation

## Abstract

**Background:**

An understanding of the nature of long Covid (LC) is evolving, with recent evidence highlighting the role of increased sympathetic activation and decreased parasympathetic response. Building upon this emerging science, the ‘Transforming Long COVID’ (TLC) programme was developed to support participants in their recovery by (i) introducing education on the neuroscience underpinning persistent symptoms (with a particular focus on the autonomic nervous system) and (ii) the development of self‐management strategies to support recovery. The aim of this study was to examine the feasibility of the TLC programme with a cohort of people significantly affected by LC.

**Methods:**

Seventeen participants took part in the 8‐week TLC programme which comprised of seven content sessions and one discussion (Q&A) session. Participants completed survey scales (investigating anxiety, pain‐related interference, pain catastrophising, sleep disturbance and fatigue) at baseline, immediately post‐programme (at 8 weeks), and retention (at 13 weeks). Participants also took part in focus group interviews to investigate their experiences of the programme.

**Results:**

Fourteen participants (82%) attended at least six of the seven TLC content sessions. Decreases in mean values over time were observed across all measures, indicating a positive (non‐significant) change. Participants reported an increase in understanding of LC, new hope for recovery, belief that they now had a realistic pathway for recovery, validation of their experiences and symptoms, meaningful improvements in function, and enhanced ability to respond to and attenuate physical symptoms. No adverse events were reported. Participants highlighted a number of programme strengths, along with some potential areas for improvement.

**Conclusion:**

The TLC programme was shown to be feasible based on engagement, adherence, acceptable completion of surveys, and no adverse events. Study findings point to the potential for this programme to be refined, trialled and evaluated with a larger sample.

**Patient or Public Contribution:**

Four people (living with LC, ME/CFS, chronic migraine and chronic Lyme, fibromyalgia, and centralised pain syndrome), who have experience of applying a recovery approach aligned with the TLC programme, acted in a PPI (Public and Patient Involvement in research) capacity on this study. In addition, the lead author has personal experience with the illness, and developing the recovery approach, which helped inform programme structure and development [1]. These individuals provided advice and guidance on the potential structure for the group programme, course duration, tool selection, and language and wording of the programme and materials. Further detail is provided in the Supplementary Materials.

## 1 Introduction

Long Covid (LC) has been acknowledged as an unprecedented burden to patients and healthcare systems (affecting an approximated 150 million people worldwide), with the long‐term impacts still to be realised [2]. The burden of LC (affecting 10%–20% of those infected by SARS‐CoV‐2 [3]) can vary from mild to profound disability, lasting for months or years [3], with significant impact on everyday functioning and quality of life [4].

Faghy et al. describe a heterogeneous aetiology of LC, representing a set of complex and interacting factors intersecting to produce a complex clinical presentation [2]. LC can result from identifiable organ injury (as detected, e.g., by pulmonary imaging or cardiac testing) which is most typically observed after severe acute Covid‐19 infection [5]. Despite a high burden of persistent symptoms in persons living with LC, abnormal findings on physical examination and diagnostic testing are reported to be relatively uncommon [5]. Donnino et al. [5] highlighted that while some reports have pointed to potential aetiologies for LC (such as capillary micro‐clots, gut viral reserves, low cortisol levels and mitochondrial dysfunction, among others), these findings have not been confirmed and/or have not been shown as the direct cause of symptoms experienced. A more recent review concludes that research into the underlying pathophysiological mechanism in LC is scarce and heterogeneous [6]. Recently, sympathetic overactivation [7, 8] and diminished parasympathetic response in LC patients have been highlighted [8, 9], suggesting a sustained impairment of sympathovagal balance and autonomic dysregulation (dysautonomia) [8, 9, 10] which could be underlying or contributing to the condition.

A conceptual model of a psychophysiologic process underpinning LC (particularly in persons with mild/moderate initial Covid‐19 infections and with no identifiable organ injury) has been posited, which authors argue explains both physical and psychological LC symptomology [5]. Psychophysiologic responses are explained as ‘*those that have physical/physiological outputs centrally mediated in the brain*’ [5, p. 338]. Similar to recent understandings of autonomic dysregulation playing a significant role in Myalgic Encephalomyelitis/Chronic Fatigue Syndrome (ME/CFS) [11, 12, 13], it is suggested that central nervous system changes develop during or shortly after acute Covid‐19 infection [5, 14]. This conceptual model has been applied successfully to treat chronic pain [15, 16], with recent research supporting the efficacy in decreasing symptom burden in people living with LC [5].

A supported self‐management programme ‘Second Arrow; Transforming Long COVID’ (TLC) evolved from an initial individual coaching programme developed by the first author, with a goal of mediating LC symptoms. Applying PPI principles, the initial programme structure was developed by the lead author through iterative conversation with four individuals living with chronic conditions, to maximise the potential acceptability and efficacy of the programme [17]. TLC is underpinned by the conceptual model of autonomic dysregulation underlying LC symptoms [1, 8, 9, 10], and LC symptoms being mediated by the central nervous system [5, 18], and builds on the work of others [5, 18, 19]. The purpose of the current study was to investigate the feasibility of the TLC programme with a cohort of Irish people living with LC.

## 2 Materials and Methods

This single‐cohort feasibility study was conducted in line with Medical Research Council guidance for the development and evaluation of complex interventions [20], to investigate progression criteria regarding evaluation design (including recruitment, data collection, retention and outcomes) and the TLC programme structure and content (including participant numbers, content and delivery, acceptability, and adherence capacity of providers to deliver the intervention). As this was a feasibility study, no formal sample size calculation was undertaken. All TLC programme participants were invited to participate, resulting in a pragmatic, opportunity‐based sample used to explore trends and experiences rather than to test hypotheses. A mixed methods design was employed, combining survey and focus group interviews.

### 2.1 Participants and Recruitment

**Figure 1:**
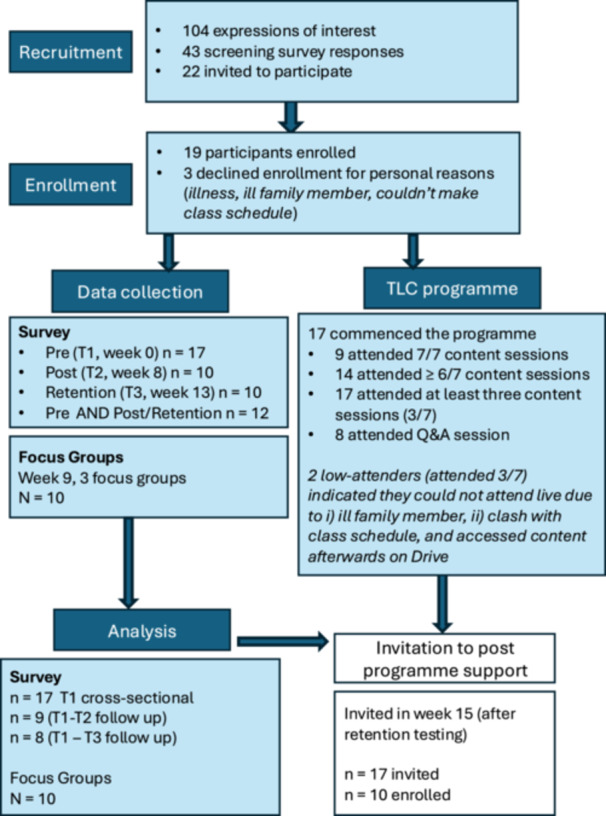
Participant flow through the study.

TLC was delivered in a personal voluntary capacity by the lead author in partnership with ExWell Medical, a national community‐based chronic illness exercise rehabilitation programme. Participants were recruited through existing ExWell mailing lists (see Supplementary Material and Figure 1). An introductory email/text was sent to all ExWell participants explaining the programme and seeking expressions of interest. Those interested were asked to provide details on confirmation of LC diagnosis, date of diagnosis, severity of symptoms, and impact of symptoms on daily life. Forty‐three participants responded. A sample of 22 was targeted as a feasible group size [5], and participants were invited based on (i) a confirmed diagnosis of LC, (ii) severity and duration of symptoms, and (iii) impact of symptoms on daily life (those with a LC diagnosis, and most severely impacted for the longest duration were prioritised).

Approval for this study was obtained through the DCU (Dublin City University) Research Ethics Committee (DCUREC/2024/167; 14 October 2024), and all procedures were performed in compliance with this approval. Respondents who were invited to join the TLC programme with ExWell were invited to also participate in the feasibility study being conducted by DCU, but were advised that their decision regarding research participation would not affect their eligibility to attend the TLC programme. Research recruitment took place between 14 and 23 October 2024. Online written informed consent was given by participants prior to data collection. Participants were advised that they could withdraw from participation in the research study at any time. The lead author, as a member of both the programme delivery and research teams, was not involved in participant recruitment or data collection and in data analysis until after the point of anonymisation. Participants were advised that the programme was not a replacement for medical treatment, and the study team did not advise on, or interfere with, treatment decisions made by participants and their healthcare teams.

### 2.2 The TLC Programme

The Second Arrow TLC programme aims to help people living with LC to develop an understanding of the potential role of the autonomic nervous system in perpetuating chronic illness symptoms and the neuroscience underpinning persistent physical symptoms and to develop strategies and apply innovative tools to aid recovery. The TLC logic model is shown in Figure 1 (detailed protocol in Supplementary Materials). Broadly, the core components were as follows: (i) Neuroscience education (including the biopsychosocial aetiology of chronic conditions); (ii) Somatic tracking and awareness tools and strategies, (iii) Self‐awareness tools and strategies; (iv) Desensitisation techniques (including visualisation/imaginal practices); (v) Emotional awareness and expression; education and strategies; and (vi) Working with thoughts; education and strategies.

TLC was delivered weekly (8 sessions October–December 2024), using the video conferencing tool Zoom. Sessions 1 – 7 (90 min each) involved neuroscience education, integration and practice of applied strategies and tools, and discussion (Q&A) opportunities. Session 8 involved a longer discussion/Q&A opportunity (approx. 1 h), delivered in two smaller groups. Weekly content was pre‐recorded in 2–3 short (15–25 min) video clips and made available (via a shared drive) to participants immediately after each session. Participants were advised (i) to join sessions in any way available to them, (ii) that there was no requirement to turn on camera or microphone (though they could do so should they wish) and (iii) if they were unable to join on a given day, or unable to join for a full session, the content would be available to them for self‐paced access on the shared drive.

### 2.3 Measures

#### 2.3.1 Survey

Participants were asked to complete an online survey at Week 0 (T1; Pre‐test), Week 8 (T2; Post‐test) and Week 13 (T3; Retention). The survey, which was emailed at each of the three time points, addressed the following items: pain catastrophising, fatigue, sleep disturbance, pain interference and anxiety (see Table 1). One open‐ended question was also asked ‘*Please list/describe any substantial lasting effects that you attribute to a Covid‐19 infection (“long Covid”)*’. Adverse events (AEs) were noted as negative clinically relevant changes to outcomes over the course of the intervention [5]. In such cases, the clinical study lead contacted any affected participant and then met with the other study clinical staff to discuss the likelihood that any AE was attributable to the programme.

**Table 1:** Outcome measures.

Outcome (and tool)	Detail	Processing
**Pain Catastrophising** (Pain Catastrophising Scale [20])	− 13 items; 5‐point scale, investigating degree to which respondents have a range of thoughts and feelings associated with pain − Cronbach's Alpha (*α*) = 0.956).	− Scores summed to give a total score. − Score of 30 representing clinically relevant level of catastrophizing [20] (categorised as present (1) or absent (0)).
**Fatigue** (Fatigue Severity Scale [21,22])	– 9 items, on 7‐point scale, investigating the extent of agreement with statements regarding fatigue over the previous week – *α* = 0.919	– Scores summed to give a total score. – Participants categorised into fatigue severity groups [23] (0–35 ‘mild/no fatigue’, 36–52 ‘moderate fatigue’ and 53+ ‘severe fatigue’).
**Sleep disturbance** (PROMIS Level 2 Sleep Disturbance Short Form [24])	– 8 items scored on a 5‐point scale evaluating the presence of sleep disturbance during the previous week – *α* = 0.844	– Scores summed to give the total score. – Raw total score converted to a T‐score and level of sleep disturbance categorised using conversion tables and categories given on the PROMIS website (<55 = None to slight, 55.0–59.9 = Mild, 60.0–69.9 = Moderate, 70+ = Severe) (nihpromis.org).
**Pain interference** (PROMIS‐Pain Interference‐Short Form 8a [25])	– 8 items, ranking pain interference with a variety of activities over previous 7 days on a 5‐point scale – *α* = 0.915	– Scores summed to give total score. – Raw total score converted to a T‐score using the conversion tables given on the PROMIS website (nihpromis.org). – T‐scores then categorised [26] (≤ 50 = within normal limits, 51–60 = mild, 61–68 = moderate, > 68 = severe).
**Anxiety** (Generalised Anxiety Disorder 7‐item scale (GAD‐7) [27])	– 7 items investigating anxiety symptoms over previous 2 weeks on a 4‐point scale – *α* = 0.901	– Scores summed to give total score. – Cut‐off value of 10 employed as an indicator of GAD [28] and categorised as present (1) or absent (0).

#### 2.3.2 Focus Groups

Following the programme, participants were invited to join one of three focus group interviews (45–60 min), with questions pertaining to overall experience, accessibility of content, perceived impact, recommendability to others, and suggestions for programme refinement/improvement (See Supporting Information).

### 2.4 Data Processing and Analysis

Employing a convergent parallel design [21], qualitative and quantitative data were analysed separately and then combined in Section 4.

#### 2.4.1 Quantitative Data

Scores for each scale were summed to give a total score (see Table 1). Normality was assessed using Shapiro–Wilk tests; minor departures were observed for fatigue and anxiety at T2. Given the exploratory aims and small sample size, paired‐samples *t*‐tests were used to explore differences in raw scores for each variable over time, with findings interpreted cautiously.

#### 2.4.2 Qualitative Data

Interviews were transcribed verbatim, and at this stage, participants were assigned pseudonyms, and minor edits were made to excerpts to hide identifiable information and for clarification which is denoted by ‘[…]’. Data analysis involved six recursive phases of familiarization; coding; generating initial themes; reviewing and developing themes; refining, defining and naming themes; and writing up [22]. Data familiarisation was enhanced by reading transcriptions in an iterative manner, while noting patterns and observations. Following best practice examples in the demonstration of rigour in Reflexive Thematic Analysis [22], three research team members discussed the generated codes and initial themes, and this collaborative approach led to richer interpretation via theme refinement [22].

## 3 Results

### 3.1 Participant Flow Through the Study and Baseline Characteristics

Participant flow is summarised in Figure 1. Participants were 58.61 ± 11.92 years (range: 31–74 years) and reported living with LC for 43.33 ± 9.83 months. Programme attendance was high, with 14 of 17 participants (82%) attending at least six of the seven content sessions and nine attending all sessions; despite some unavoidable absences (see Supporting Information). Seventeen participants completed the survey at T1, while ten participants completed the survey at T2 and ten completed at T3. Twelve different individuals completed the survey at T1 and T2, *or* T1 and T3. Ten participants participated in the focus groups.

### 3.2 Survey Data

Table 2 presents baseline participant demographics, along with LC symptoms, and raw and categorised baseline outcome measures.

**Table 2:** Participant demographics and outcome measurements at baseline (*n* = 17).

	Frequency	Mean (SD)	Range
Age (years)		59 (12.166)	31–74
Gender	14 female, 3 male		
Duration living with LC (months)		43.94 (9.782)	26−59
**Self‐reported primary symptoms of LC (open Q)** *			
Fatigue	16 (94%)		
Pain (Joint/Muscle/Neuropathic/Chest)	9 (56%)		
Brain fog/Cognitive Impairment	7 (41%)		
Post Exertional Malaise (PEM)	6 (36%)		
Headache and/or Migraine	3 (18%)		
Sleep Issues (including Insomnia)	3 (18%)		
Digestive issues	2 (12%)		
**Survey items raw scores**			
Pain Catastrophising Scale		13.2 (11.614)	0−39
Fatigue Severity Scale		53.94 (9.71)	34−63
Sleep Disturbance		27.24 (6.16)	15−39
Pain Interference		24.76 (7.37)	12−40
Generalised Anxiety Disorder (GAD‐7)		7.41 (5.292)	0−21
**Diagnosis/Category per survey response (*n*)**			
Pain Catastrophising Scale	2 Clinically relevant		
Fatigue Severity Scale	4 Moderate, 12 Severe		
Sleep Disturbance	4 Mild, 7 Moderate, 1 Severe		
Pain Interference	7 Mild, 9 Moderate, 5 Severe		
Generalised Anxiety Disorder (GAD‐7)	7 Possible presence GAD		

* Open question; the terms presented here are those used by participants.

Table 3 presents mean (SD) scores for outcomes at T1 and T2, and T1 and T3, with *t*‐test confidence intervals and *p* values. Positive mean changes (i.e., decreases in scores) were recorded across all outcome measures over time, with the exception of sleep disturbance which showed a mean increase of 1.25 from T1 to T3. With the exception of Pain Interference from T1 to T2, where a significant positive change was recorded (*p* = 0.039), changes were not statistically significant.

**Table 3:** Mean (SD), 95% CI, and mean difference for outcomes at T1, T2 and T3 (*n* = 12).

	Pain catastrophise	Fatigue severity	Sleep disturbance	Pain interference	Anxiety
**Mean (SD) T1–T2 (*n*** = **9)**					
T1	14 (11.02)	52.89 (9.93)	29.22 (5.71)	24 (8.63)	7.22 (6.14)
T2	10.67 (12.53)	40.33 (18.62)	26.78 (7.71)	19 (10.11)	5.78 (5.78)
**Mean (SD) T1–T3 (*n*** = **8)**					
T1	14.43 (12.71)	54.63 (8.417)	26.63 (7.46)	27.63 (6.97)	8.38 6.854
T3	13.29 (9.09)	53.63 (7.689)	27.88 (5.22)	23.5 (11.07)	6.75 (2.92)
**Mean difference**					
T1–T2 *n* = 9	−3.33 (95% CI: −6.4, 13.06), *p* = 0.452	−12.56 (95% CI: −0.71, 25.83), *p* = 0.061	−2.44 (95% CI: −2.79, 7.67) *p* = 0.312	−5 (95% CI: 0.31, 9.69) *p* = 0.039	−1.44 (95% CI: −4.87, 7.76) *p* = 0.612
T1–T3 *n* = 8	−1.14 (95% CI:−10.93, 13.22) *p* = 0.825	−1 (95% CI: −8.32, 10.32) *p* = 0.807	1.25 (95% CI: −5.17, 2.665) *p* = 0.475	−4.13 (95% CI: −1.54, 9.79) *p* = 0.129	−1.63 (95% CI: −2.17, 5.415) *p* = 0.344

Table 4 presents individual trajectories in outcome categories over time; 16 positive and 2 negative changes were recorded over the three time points.

**Table 4:** Individual trajectories in outcome categories across time.

Participant	Time points	Pain cat	Fatigue	Sleep	Pain int	Anxiety
1	T1 T2 T3	↓ →	→ →	↑ ↓	→ →	→ ↓
2	T1 T2 T3	→ →	→ →	↑ ↓	↓ ↑	→ →
3	T1 T2	→	↓2	↓2	↓	→
4	T1 T3	→	→	→	→	→
5	T1 T2 T3	↑ ↓	↑ →	→ →	→ →	↑ ↓
6	T1 T2 T3	→ →	↓2 ↑	↑ ↓	↓ →	→ →
7	T1 T3	→	↓	↑	↓	→
8	T1 T2	→	→	↓	→	→
9	T1 T2	→	↓	↓2	→	↓
10	T1 T3	→	→	→	→	→
11	T1 T2	→	→	→	↓	→
12	T1 T2 T3	→ →	↓ ↑	↓ →	→ →	↓ →

↓ = improvement by one category level.

→ = no change in category level.

↑ = deterioration by one category level.

↓2 = Improvement by two category levels.

Two participants recorded increases in outcome measures (fatigue and/or pain) and were followed up by the clinical study lead. One participant attributed their change in scores to a new medical diagnosis unrelated to LC, and the second attributed theirs to a flare of their LC. Neither participant attributed their change to the intervention.

### 3.3 Focus Group Data

Ten participants took part across three focus groups. Three themes relating to participants' experiences of the pilot programme were constructed from the data; Table 5 presents sample quotes for each theme (or sub‐theme where relevant), which are elaborated in the following text.

**Table 5:** Themes, sub‐themes and sample quotes.

Theme	Sub‐Theme	Quote
Theme 1: The programme provided validation, hope and meaningful change	Sub‐theme 1.1: Hope: A light at the end of the tunnel	*…I've been trying to understand what's going on in my body and like feeling like you've no like hope…. You're not getting that information from doctors and stuff. Like I've been attending the long Covid clinic in [specific hospital], and, like the doctor side of it has just been very lacklustre…. You're coming to them, and I try to not be emotional about it [experiences of long COVID], and just give the information to them. I'd just be met with like “Keep doing what you're doing” like no explanation of what's going on and that just adds to the stress and the fear … it's such an awful loop.* Cara
		*Grace: ‘I'm in my late 60s, and I just felt that I was entering into a very miserable old age, that my world was getting smaller and smaller and smaller, because everything I did ended up in pain that didn't go away for a long time, and that was corrected almost at the beginning, that when I understood the mind body connection, and that the pain wasn't dangerous, and that the pain was my brain trying to keep me safe, and my brain making a big mistake that just, oh…. that just, was my launching pad to a happy and fruitful old age I feel.* *Facilitator: ‘If you don't mind me saying, because it won't go on the audio, I can even see as you're talking there, you can see the smile kind of come across your face as you started talking about that kind of moment…* *Grace: ‘Yeah, you can't see it, but my eyes have watered up with the relief.* *Marie: ‘Yeah, I understand you exactly, Grace, because that was the way I felt the last four years, “the pensioner.” And it's like, honestly, is this what I have to live with? You know, and…. and, and the world getting smaller … and this has given me new hope.*
	Sub‐theme 1.2: The validation of a shared journey	*To know that you're not going mad as well, … there was a group of people there that all had symptoms.* Marie
	Sub‐theme 1.3: Meaningful wins	*I really wanted to be able to walk up that driveway, walk up those steps into the house and go in and say hello to my folks, and I put that in my head that I was going to do it; and I did it without repercussions.* Jack
		*Not only the sun, but the sun reflecting on other cars, is like kind of like spears going into my eyes, you know, going into my brain. And I started feeling like my neck tensing up. And I started feeling like my stomach tightening. So I said, You know what? Let me start doing the affirmations. And you know, telling my brain that it was safe and everything. And I actually started feeling everything starting to calm down a little bit, you know, and it was just kind of like the low level of pain, not the like excruciating pain, you know.* Deirdre
Theme 2: Engaging and supportive programme with varied individual experiences	Sub‐theme 2.1: Specific programme components participants found beneficial	*One of the things that [the coach] mentioned, and it triggered in me. I was in rehab after COVID, and I couldn't do things until I could see myself doing them in my head. And [the coach] referred to that, and that was just I had never thought of applying it to my everyday life. So that was absolutely brilliant.* Michael
		*“…like challenging your brain and saying to your brain. Brain. I'm fine now. You don't have to worry. You can just take a rest. I'm safe. I'm in a safe place.” Even those words alone, and even that little bit of input alone, can totally de‐stress me and relax me and everything. I find the course absolutely amazing.* Aine
		*She's very calm and she just has a wonderful like personality, voice, whatever that package is, that it made you listen to her. You know, the way sometimes when people talk, you know, you can drift away from them, you lose concentration. But I found with her, she was able to hold my attention so, and because she has also experienced, you know, COVID, that's reassuring, that there's somebody that can battle through it*. Marie
	Sub‐theme 2.2: Conflicting participant needs: Individual challenges with the programme	*You don't know which one [technique] suits me, or Marie or Grace or Michael, So there's a lot there for us to all go back to [in the drive folder] and pick our own bits. So I think there's a lot in it.* Paul
		*I found it overwhelming. To be honest my concentration isn't very good at the moment, and I certainly think an hour and a half of a session was too long … but like I'm struggling with, I'm having cognitive issues, and I have very bad fatigue. And I just found so much coming at me.* Aisling
Theme 3: Strengthening and extending the programme for future delivery		*There's no doubt that [the coach's] long COVID experience added weight to her words, but I suppose I feel people can be trained to present a course, and learn empathy and compassion like [the coach] exhibited.* Grace
		*I think a breakout group after the session would give people more of a chance to kind of connect and talk and like, express what they're going through and what they're feeling and what they are taking from the course.* Cara

#### 3.3.1 Theme 1: The Programme Provided Validation, Hope and Meaningful Change

Though the emphasis within focus groups was on participants' experiences of the TLC programme, throughout the conversations all participants gave insight into their personal experiences of living with LC. While participants shared willingly, the tremendous weight and impact of LC was evident. The impact of these symptoms was described as ‘*Alive but not living*’ [Jack] by one participant, and others shared detail on their previous experiences of treatment.

You felt like you were the only one, and you were going mad, and that you're making it up, or it was all in your you know head, just, you just didn't know how to fix it, and there was no medical drug you could take or quick fix.

Paul

This first theme highlights a transformative impact, both physically and emotionally, as a result of taking part in the TLC programme; represented by three distinct sub‐themes which describe the change in participant's perceptions and outlooks.

##### 3.3.1.1 Sub‐Theme 1.1: Hope: A Light at the End of the Tunnel

The first of these sub‐themes paints a picture of life with LC wherein all participants felt they had been at a dead end. Many had faced a lack of understanding from others, little to no relief in symptoms from treatments, and struggled to see how their conditions, and their lives more broadly, were going to improve. The feeling expressed by participants with regard to their LC healthcare experience was one characterised by a lack of information and an endless loop. Within each of the focus groups, it became clear that one of the biggest impacts of the programme was a new sense of hope for recovery, which was singled out by many as the most beneficial aspect of the programme.

Participant's sense of new hope was presented with a sense of realism. While the programme had enabled them to be hopeful that recovery was possible, there was an understanding that it was going to take time to get back to where they were prior to Covid. Jack, like many other participants, had been forced to medically retire as a result of LC, which had contributed to a shift in identity and purpose. For him this programme was:

…fantastic, absolutely life changing. Before the course, I was just totally miserable, and I couldn't function so this not only gave us hope, but showed us a path to kind of follow. And I've genuinely improved.

Jack

The metaphor of the path or the journey was used in all focus groups. Participants recognised that their recovery from LC was an individual experience that could take a long time, with ups and downs, *‘It's just been the start of a journey, and not the end of it’ (Paul)*. Critically, the frequent use of the terms ‘path’ and ‘journey’ conveyed that the participants could not only see a future for themselves, but they saw a positive future for themselves.

##### 3.3.1.2 Sub‐Theme 1.2: The Validation of a Shared Journey

Many found the group experience of the pilot programme to be a valuable way to share experiences, knowledge and resources with others in a similar situation. Or, as Cara put it; ‘*Just hearing people's stories and misery, and laughing with them’.* The shared group structure helped to validate experiences and provided reassurance that they were not alone in their struggle with long‐term symptoms.

##### 3.3.1.3 Sub‐Theme 1.3: Meaningful Wins

Different ‘meaningful wins’ were cited as positive outcomes from taking part in the programme. One participant described how he had not been able to walk up the driveway to his parents' house for a number of years, and overcoming this with the aid of tools and strategies learned on the programme had been a key moment for him. Another participant articulated the benefits of an ability to respond differently to migraine symptoms using tools learned on the course. Meaningful wins were founded on each individual's personal values and goals, such as being able to make dinner, tidy the house, sit in front of a computer, or being able to go for a walk. Not all goals had yet been achieved, but participants illustrated that they saw this as a journey (see Sub‐Theme 2.1) and, critically, felt that their goals were achievable now, in that ‘*little bit of hope at the end of the tunnel’ (Deirdre)*.

#### 3.3.2 Theme 2: Engaging and Supportive Programme With Varied Individual Experiences

The majority of participants (9/10) believed that the programme could be a huge benefit to other people living with LC, with one participant describing difficulties engaging with the volume and pace of content due to cognitive and concentration challenges. Despite the acknowledgement of the difficulties in considering and catering for each individual's personal experiences with LC, participants spoke of different programme components (such as activities, resources, strategies, techniques and logistical considerations), that they felt contributed to the positive impact of the programme.

##### 3.3.2.1 Sub‐Theme 2.1: Specific Programme Components Participants Found Beneficial

Speaking about the early sessions, participants voiced a sense of relief learning about the mind–body connection and an increase in self‐awareness of what was happening in their bodies.

It's amazing that this course helps people to understand … like Jack said, like the chain of events, the links of everything, and how to kind of bring that awareness to it, and to stop it in it's … well, not … we can't always be able to stop it, but to like calm it down in its tracks, and not let it go to the moon like, you know. I think that's amazing.

Cara

Participants spoke of a number of specific exercises they learned in the sessions that they found beneficial, recalled as: affirmations, meditation, reflective writing, somatic exercises, self‐talk and visualisation. Many participants spoke of the benefits they got from using the somatic guided audio tracks provided; *‘Her voice, that is particularly, I found very soothing, … and the little tracks I found particularly effective’ (Deirdre)*. A new ability to use ‘self‐talk’ to calm and relax their responses in the face of challenging symptoms was articulated by many participants.

The exercises and new knowledge encountered on the programme not alone relaxed, but empowered participants; *‘I feel I have…. Maybe I've taken a little bit more control…’ (Bridget)*. The pacing and evidence‐based delivery of the programme for individuals living with chronic fatigue and ‘brain fog’ (term used by participants) instilled a confidence in the activities. One participant spoke of appreciating the repetition: *‘If you say that [it's repetitive], it sounds negative, but it's actually very positive, because part of my condition is memory loss’ (Paul)*. Many participants cited the online folder of resources, including guided audio tracks, as a valuable tool.

With the session content recordings and other supplementary resources available on the shared drive online, participants acknowledged that there was quite a lot of information, but the majority felt that access to this information was beneficial and that they were able to ‘*dip in and out*’ (Michael). Acknowledging that the programme had come to an end, one participant mentioned that they were going to complete the whole course again on their own with the online content, and another added; ‘*I will listen to those sessions that are up on the folder, and I get a great deal of comfort out of that*’ (Paul). Practically, participants spoke of the comfort in being able to have the camera off during sessions and the ease of having both live and recorded sessions; ‘*…if it was too much, you could do it at your own pace during the week’ (Grace)*.

Finally, the facilitator was cited as a significant contributing factor to the felt success of the TLC programme by participants. Many participants found her voice to be a soothing comfort in the sessions and recordings. The fact that the facilitator had also had LC was another notable factor underlined by participants. The authenticity, relatability and the understanding of the lead facilitator was discussed within all focus groups.

…has been through this herself, and all of this information that she's passing on is based on a lot of the research that she did to help herself get better. So she's not a clinician who's one removed from it, like I go see a neurologist…. They don't have this condition, and they never had it. They understand it, maybe up to a point, but the fact that [the facilitator] is the person pulling all this [together] that was, I think, extremely important as well. It was more believable, … it just, it just added something to it for me, and she could refer back to her own personal circumstances, which some of us were able to relate to.

Paul

##### 3.3.2.2 Sub‐Theme 2.2: Conflicting Participant Needs: Individual Challenges With the Programme

This sub‐theme describes how different individual challenges impacted participant engagement and the subsequent perceived effectiveness of the programme. For example, as described in Sub‐Theme 3.1, while a majority of participants found the course accessible between the live and pre‐recorded content, valued content repetition, and the access to supplementary resources following the weekly sessions, one participant found the content overwhelming due to difficulties in concentration. While most participants cited the breadth of content and tools as reassuring, this breadth had the opposite effect for this participant. Discussions across the focus groups highlighted some varied opinions regarding the timing, content, length and frequency of the sessions, but participants also cited an awareness that what might work best was often down to varied individual preference.

Maybe just the time…. I mean, if that [the live sessions] could be done sort of afternoon time … and again an afternoon might not suit everybody. You know everybody's different.

Jack

#### 3.3.3 Theme 3: Strengthening and Extending the Programme for Future Delivery

Given the challenge of retaining attention while living with brain fog and fatigue, some participants called for individual sessions to be shorter than 90 min, but then acknowledged that it was important not to lose the opportunity for guided somatic practices threaded through the sessions ‘*I think if you shorten the sessions, you wouldn't be able to include the meditation sessions, and they were a big part for me’* (Michael). In terms of duration of the programme (8 weeks), many wanted it to continue, or at least, have the opportunity for follow up sessions in the future ‘*…a follow up class once a week, or even once every 2 weeks … just somatic practice and different things’* (Aine).

Another participant mentioned the possibility of an online support group to be able to ask questions, but to also challenge the sense of isolation people felt when living with LC. In line with this, the limited opportunity for group interaction during the course was highlighted as an area for improvement, ‘*We were a part of a group. But I wouldn't be able to tell you the names of the people that were in’* (Aisling). Maire mentioned wanting to be able to introduce herself in the earlier sessions, but Michael highlighted the challenge of this ‘*…there may be a discomfort about kind of identifying yourself’* (Michael), while Cara noted ‘*I was just basically nonverbal and too fatigued to kind of interact like while the course is going on’*. A group chat was suggested, as well as breakout rooms, which one participant felt may combat the fear of talking in a larger group.

While the role of the lead facilitator, and the peer support she offered, was considered to contribute significantly to the success of the programme, participants acknowledged that it would not be feasible for this one facilitator to deliver the programme to everyone that needs it. It was suggested that *‘…there could be an input, if possible, at some stage in the course from somebody who has gone through the course?’* (Michael). Crucially, participants recognised that for the programme to be continued and developed ‘*more doctors need to know about this’* (Deirdre), ‘*I want everyone to have it, and everyone to be on it, and everyone to see it’* (Aine).

## 4 Discussion

Evidence supporting the use of group compared to individual interventions is varied and in many cases unknown [23], and the importance of applying a mixed methods approach to evaluation has been underlined [23]. The question of whether TLC could be translated and delivered in an acceptable format in a group coaching environment was an important one to investigate in this study. Critically, this feasibility study combined PPI with a person‐based approach, employing qualitative research to gather an in‐depth understanding of the experiences and perspectives of people living with LC, who experienced the intervention programme from a fresh perspective [24]. The exploratory findings support the potential for effective and impactful delivery within a group environment. The anticipated challenges of not being able to individualise the coaching approach and strategies and refine content to meet specific individual preferences within the group environment were recognised by participants. Consistent with others, however [25, 26], the additional benefit the group environment brought in offering a sense of belonging, identity and support through a ‘shared journey’ was highlighted by participants as a very worthwhile benefit.

Most existing interventions for LC focus primarily on physical rehabilitation, exercise and standard psychological approaches and are largely oriented towards symptom management [4, 27, 28, 29, 30]. The TLC programme is centred on neuroscience‐informed education and self‐management strategies targeting autonomic dysregulation and central processing. While research applying this conceptual model to LC remains at an early stage, Donnino et al. [5] reported improvements across multiple outcome domains using a similar approach. Systematic reviews suggest that existing LC interventions typically demonstrate small to moderate effects [4, 27, 28, 29, 30]. Although the small sample size in the present study precludes formal estimation of effect sizes, the observed patterns of improvement across several outcomes and time points, in a cohort of people who have lived with persistent LC symptoms for an average of 44 months, suggest potential clinical relevance and support further investigation in adequately powered trials.

The range of LC physical symptoms reported by participants in the current study map with those previously reported in the literature [31, 32, 33, 34] and, consistent with others [4], the qualitative findings underline the impact these symptoms have on participants' physical, cognitive and mental functioning, as well as quality of life. The TLC programme was developed with a goal of improving the quality of life and reducing the symptom burden of people living with LC. Findings of the current study support the potential for the programme in this regard. Although the understanding of autonomic mechanisms and the development of regulation strategies were not measured using specific quantitative instruments in the current study, these TLC programme objectives were reflected clearly in the qualitative findings. Participants frequently described gaining a new understanding of the mind–body and autonomic processes underpinning their symptoms, alongside learning practical strategies to calm physiological arousal (such as somatic awareness, self‐talk, visualisation and relaxation techniques). These changes were articulated as increased confidence in self‐management and perceived control over symptoms, suggesting that the programme's core theoretical and practical aims were meaningfully achieved.

Attendance rates across the 8‐week programme along with qualitative data overwhelmingly point to the acceptability of the programme to participants. Participants articulated a desire for TLC to be extended so that they could remain involved for longer‐term support and it may be offered to more people. The absence of adverse events and high levels of engagement in this study are consistent with findings from other psychologically and behaviourally informed LC interventions [5, 35]. Following MRC guidance [20], findings of this study support progression of TLC to an appropriately powered evaluation trial.

Outcome measures chosen for the current study align with the reported symptom burden of those living with LC [31, 32, 33, 34]; however, other measurements are worthy of consideration for inclusion in an evaluation trial. For example, in the context of LC as a condition mediated by ANS dysfunction [8, 9], the inclusion of indicators of ANS function (such as heart rate variability) may be very important. In addition, qualitative findings suggest that benefits of TLC are linked not only to symptom‐focused outcomes, but also to increased validation, hope and confidence in recovery. Optimism and hope have been identified as important factors influencing healthier behaviours and better outcomes for people living with chronic conditions [36] and are associated with higher self‐esteem and lower rates of depression [37]. A newly found hope for recovery was strongly articulated by participants in the current study and is likely linked to a focus on ‘belief in recovery’ threaded through the programme. This was supported by the programme education components (with scientific evidence threaded throughout) that explained how the mind and body can interact to perpetuate physical symptoms and how we have the capacity to develop tools and skills to moderate this. Many participants highlighted this new knowledge as an important factor in their new found hope, which was supported by the small but ‘meaningful wins’ they personally experienced as they applied the tools and strategies. Recognised as important determinants of engagement and outcomes in chronic illness management [36, 37, 38, 39], future research should consider the inclusion of measures such as validation, hope and confidence—mechanisms through which the TLC programme may exert it's effects.

Aligned with the above, treatment expectations have also been identified as an important factor predicting treatment outcome across many physical and psychological conditions [38, 39]. Considered the central mechanism behind placebo and nocebo effects, a person's expectations can induce relevant physiological changes that influence treatment response [39, 40]. Indeed, Kube et al. [41] explain the role that dysfunctional expectations can have in terms of ‘cognitive immunisation’—a tendency to evaluate novel incoming information in a negative manner to fit with initially negative expectations. They advocate that for chronic illnesses categorised by persistent physical symptoms (such as LC)—a focus should be moved away from the causes of the symptoms, to the sustaining mechanisms that prevent symptoms from subsiding spontaneously [41]. This is an approach which aligns with the principles of the TLC programme and proffers treatment expectations as both an important goal, as well as another potentially important outcome measure (e.g., the Tex‐Q [39]).

In the current study, survey completion declined at post‐intervention and retention time points, despite high levels of programme attendance. This pattern likely reflects the cognitive fatigue, fluctuating symptoms and limited capacity commonly experienced by people living with LC [4, 31]. It is worth noting that the TLC study recruited participants who had been most severely impacted by LC for a prolonged period, and programme access was offered *regardless* of engagement with study measures. This decision was made on ethical grounds; given the participant profile, requiring additional cognitive tasks (such as survey completion) may have presented substantial burden to many. Similar challenges with survey completion were reported in another LC feasibility study which adopted this approach [35], whereas a study which enrolled participants simultaneously into both the LC intervention and research protocols reported higher completion rates [5]. Together, these findings highlight the need to balance ethical considerations with data collection demands and to explore alternative or simplified outcome assessment strategies in future trials.

A further ethical question to consider is whether or not to control an evaluation trial with this population, a conundrum that is not new to such research [42], with the need to weigh and balance competing ethical objectives of trial design for each trial independently being well understood [43]. A carefully controlled trial brings important validity to study findings. A self‐controlled design, however, such as that used by Donnino [5], may be most appropriate for a study population such as this, where symptoms have persisted for a significant period of time prior to the study (range: 26–59 months) and therefore ‘*would be unlikely to experience a marked improvement in symptoms in a short period of time without intervention*’ [5, p. 344]. While a self‐controlled design [44] may overcome ethical challenges faced, such as withholding a treatment from those that may need it [42] or placing a burden on control participants [10], it requires a very careful consideration of study design and analysis.

### 4.1 Strengths and Limitations

The strengths of the current study include the mixed methods approach and the inclusion of retention follow‐up measurement. The inclusion of PPI in programme and study design is another notable strength. Limitations include the relatively small sample size and low completion rates for the survey at post and retention testing. Additionally, as the recovery approach taken within the intervention programme was communicated with participants during the recruitment process, it is likely that people open to this approach self‐selected to participate, which may limit generalisability due to selection bias. The self‐reporting of outcomes may be considered a limitation, it is mitigated however by the use of validated survey tools. Finally, as the entire programme was delivered by one facilitator/coach, we cannot determine whether it would be similarly received and effective if delivered by another facilitator.

## 5 Conclusion

The need for intervention programmes to help alleviate symptom burden for those living with LC is well recognised [2, 5, 10, 45]. The potential for programmes such as TLC, which considers autonomic dysfunction as an underlying mechanism and takes a biopsychosocial approach to recovery, without replacing other healthcare supports, is considerable [1, 5, 45]. Study findings suggest the TLC intervention is acceptable and feasible. A fully powered trial examining the effectiveness of the TLC programme in conjunction with normal care is warranted.

Please see this paper's Supplemental Materials file for further details.

## Author Contributions

**Sarahjane Belton:** conceptualisation, formal analysis, methodology, project administration, writing – original draft, writing – review and editing, visualisation, validation, resources, investigation. **Hannah Goss:** data curation, formal analysis, methodology, writing – review and editing, investigation. **Enda Whyte:** data curation, writing – review and editing, investigation. **Noel McCaffrey:** conceptualisation, writing – review and editing. **Sophie Gibney:** data curation. **Kate Sheridan:** conceptualisation, data curation, formal analysis, methodology, writing – review and editing, investigation.

## Funding

The authors have nothing to report.

## Ethics Statement

Approval for this study was obtained through the DCU (Dublin City University) Research Ethics Committee (DCUREC/2024/167; 14 October 2024), and all procedures were performed in compliance with this approval.

## Conflicts of Interest

The authors declare no conflicts of interest.

## Supporting information

Supporting File:

## Data Availability

The data that support the findings of this study are available on request from the corresponding author. The data are not publicly available due to privacy or ethical restrictions. Quantitative data will be made available upon reasonable request to the lead author.
