# Supplementary material for: ‘I Want Everyone to Have It, and Everyone to Be on It’: A Feasibility Study of the Transforming Long Covid Intervention
Source: Health Expect. 2026 May 3;29(3):e70681. doi: 10.1111/hex.70681 (PMC13136607; doi:10.1111/hex.70681)
Supplement: Supplementary file 1 — Supporting File: [file HEX-29-e70681-s001.docx]

**“I want everyone to have it, and everyone to be on it”: A feasibility study of the Transforming Long COVID (TLC) intervention**

# Supplemental Material

**Table of Contents**

[Supplemental Material 1](#_Toc221262525)

[TLC Programme Logic Model 1](#_Toc221262526)

[TLC programme development 2](#_Toc221262527)

[TLC programme study recruitment 2](#_Toc221262528)

[Focus Group Question Schedule 2](#_Toc221262529)

[Participant attendance at TLC sessions 2](#_Toc221262530)

[Intervention overview 3](#_Toc221262531)

[Reported use of Intervention resources 6](#_Toc221262532)

[Tidier Checklist 7](#_Toc221262533)

## TLC Programme Logic Model

## TLC programme development

The programme was developed and delivered by the first author; an academic expert in physical activity, physical literacy and health literacy for health and wellbeing (PhD), and a certified mind body coach, meditation teacher, and ‘Freedom From Chronic Pain’ practitioner. The first author managed her own recovery from long COVID (LC) by developing and adopting this approach (Belton & Sheridan, 2025), and has since worked with others living with LC and other chronic illnesses on a 1:1 basis, coaching them in their recovery. Four of these people acted in a PPI (Public and Patient Involvement in research) capacity on this study, providing advice and guidance on tool selection, and language and wording of the programme and materials, iteratively over the course of programme development and delivery.

## TLC programme study recruitment

TLC was delivered in a personal voluntary capacity by the lead author in partnership with ExWell Medical, a national community-based chronic illness exercise rehabilitation programme. All people attending ExWell have been referred by a healthcare professional, and deemed appropriate for participation in their programmes. Participants in TLC were recruited through the existing ExWell medical mailing list; 104 responses were received following an initial invitation from the ExWell Medical Director (co-author NMcC) for expressions of interest in attending a tailored LC programme. A more detailed email (detail in Supplemental Materials) was then sent to these 104 respondents, explaining the approach that would be taken in the TLC programme, including some information on ANS dysfunction, a linked podcast of an international medical expert speaking on a mind-body approach to recovery from LC, and practical information on programme duration and delivery.

## Focus Group Question Schedule

- How would you describe your experience of participating in this programme?
- Did you find the content accessible and understandable?
- What for you was the most beneficial aspect?
- What was the most challenging aspect?
- What is your outlook for the future now?
- Would you recommend this programme to others that have long Covid? If so why, if not, why not?
- Do you have any recommendations for how the programme could be improved if it were to be offered to future groups?

## Participant attendance at TLC sessions

Table S1 below gives an overview of the spread of attendance across the 8 weeks of the programme.

**Table S1 Participant attendance across the programme**

| **Session** | **1** | **2** | **3** | **4** | **5** | **6** | **7** | **8 (Q&A)** |
| --- | --- | --- | --- | --- | --- | --- | --- | --- |
| **Attendance** | 17 | 15 | 15 | 17 | 14 | 17 | 13 | 8 |

A total of 9 participants attended all 7 content sessions, 6 attended 6 sessions, 1 attended 4 sessions, 1 attended 3, 1 attended 2 sessions, 3 of those invited did not subsequently join the course. One of these non-attenders contacted the course leader indicating that due to an unwell family member. Another indicated they could not attend due to a clash with another activity they were engaged in, but that they would be accessing the content weekly online.

## Intervention overview

The TLC programme aims to support people living with LC to develop an understanding of the potential role of the autonomic nervous system in perpetuating chronic illness, the neuroscience underpinning persistent physical symptoms (including predictive processing), and to develop strategies and apply innovative tools to aid recovery. Fundamental to the programme was the work of (Schubiner, 2022), who proposes that many ongoing symptoms of disorders such as long COVID can be caused by complex changes to the neural circuits of the central nervous system

The programme was delivered weekly over 8 sessions between October and December 2024, using the online video conferencing tool Zoom. Sessions 1 – 7 were 90-minutes long and involved a combination of theory, applied practices, and question and answer (Q&A) opportunities. Session 8 involved a Q&A opportunity (approx. 50 minutes), offered in 2 smaller groups. Weekly content was pre-recorded, and made available on a shared drive for participants to access after the class. Participants were advised to join the sessions in any way that they felt they could, that there was no requirement to turn on a camera, or a microphone, though they would be invited to do so. They were advised also that should they be unable to join on a given day, and similarly if they could only join for a portion of the class, that the content would be made available afterwards for them.

**Programme aim**:

To offer people living with long COVID education, and innovative tools and strategies to aid recovery from symptoms of long COVID.

**Programme Objectives:**

- To support people with long COVID develop an understanding of the role of the autonomic nervous system in perpetuating chronic illness, and the neuroscience behind neural circuit disorders
- To introduce tools and strategies to calm autonomic arousal
- To introduce tools and strategies to aid development of new neural circuits; including somatic awareness, self-awareness, and emotional awareness strategies
- To introduce tools to aid reengagement with daily life and physical activities
- To help participants develop confidence and autonomy in guiding their own recovery
- To provide a climate and environment that fosters hope and empowerment

**Core Pillars**

- Neuroscience education
- Somatic tracking and awareness
- Self-awareness
- Imaginal exposure and gradual reengagement
- Emotional awareness and expression
- Working with thoughts

**Information provided prior to Session 1**

Participants were advised at the outset that the programme would entail both education and coaching, with a view to helping them navigate their recovery journey. Coaching was explained as a collaborative process, with an ongoing relationship between the coach and the participants. The role of the coach was explained as one of helping participants to develop the tools, skills, confidence, knowledge and awareness needed to create the change they want in their health and life. It was explained that it is a strengths based, forward-looking, and collaborative relationship, and that in order for the relationship to be productive and effective, both coach and participants need to engage as fully as possible in the programme.

**Session 1**

This first session has the goal of introducing participants to the relationship between the brain and the body, and the significant role the brain plays in our normal daily experience. Examples are given from the medical literature of the brain making predictions based on information available, but making mistakes (Schubiner, 2022). The concept of neuroplasticity is introduced, and the role neuroplasticity can play in perpetuating persistent physical symptoms is discussed (Gordon & Ziv, 2021; Schubiner, 2022). Goal setting is discussed. Participants are introduced to a breathwork exercise as a self-regulation tool, are also lead through a somatic awareness exercise, and given access to an associated audio track after the session.

**Session 2**

Participants are introduced to the ‘FIT assessment criteria’ developed by (Schubiner, 2022) which allows people to self-determine the extent to which symptoms they are experiencing may or may not be driven by neural circuits. The role of the autonomic nervous system in activating a ‘fight or flight’ stress response, and the role and implications of a fight or flight response are explained (Gordon & Ziv, 2021; Schubiner, 2022). Participants are lead through a Somatic Tracking practice (Gordon & Ziv, 2021), and given a guided audio track to use in their own time.

**Session 3**

‘Brain talk’ is introduced as a strategy which can be used in real time to rewire neural circuits, and reassure the brain of the safety of symptoms or situations experienced (Gordon & Ziv, 2021; Schubiner, 2022). Scientific research demonstrating that use of self-affirmations produces physical changes in brain regions is presented (Cascio et al., 2016). Polyvagal theory is introduced (Dana, 2020; Porges, 2017), it’s relevance to neural circuit disorders/ANS dysfunction is explained, and implications for how we can apply the theory in our approach to recovery form long COVID is discussed. Participants are lead through a somatic awareness body scan practice.

**Session 4**

The term Nocebo is introduced and explained, and the implications for long COVID symptom persistence is discussed. The power and importance of fear and beliefs is discussed, and expanded upon with regard to physical and other daily activities people wish to reintegrate to their lives. Visualisation is introduced as a strategy which can be used to reduce the fear associated with an activity, and help to develop a capacity to slowly reintegrate activities into our daily lives. Extended exhale breathwork and somatic grounding strategies are introduced as a way of calming ANS arousal response. Participants are introduced to, and lead through, the key steps for successful and impactful imaginal exposure practice.

**Session 5**

The role that habitual thought patterns can play in reinforcing neural networks is discussed, and strategies to identify and respond to thought patterns are introduced. The role of emotions in also reinforcing neural networks is recapped, and further discussed. The three levels of mind are explained, and a model demonstrating how emotions can reinforce physical symptoms is introduced (Donnino et al., 2023; Gordon & Ziv, 2021; Schubiner, 2022). The importance of learning to notice, feel, and allow emotion is discussed. Expressive writing is discussed as a tool that may be of benefit in opening to and allowing emotions. Following the session participants are given a Befriending Emotions guided audio track, a Befriending Sensations guided audio track to use in their own time. They are also given a guideline for Expressive Writing, and a guideline for application of a strategy for working with habitual thought patterns.

**Session 6**

The importance of ‘outcome independence’ (Gordon & Ziv, 2021) is reinforced, with strategies for allowing and responding to pain or other symptoms as they arise being recapped and underlined. Visualisation and imaginal re-engagement strategies are recapped, and the potential of, when ready, gradually reengaging with an activity that has been out of reach (e.g. household, social, or physical) is introduced, and the importance of very gradual paced progression underlined. Participants are lead through a ‘Best Possible Self’ visualisation exercise (adapted from (Peters et al., 2010)). The role and importance of beliefs is discussed. Following the session participants are given a sleep focussed somatic awareness guided audio track to use in their own time.

**Session 7**

The inevitability of flare-ups, set backs, and relapses as we progress with recovery are discussed (Gordon & Ziv, 2021), and the potential for viewing these as an opportunity to apply the various tools and strategies to do some very powerful rewiring of neural circuits is explained The need to move very slowly and gently in progressing activities is reinforced; gently stretching boundaries, rather than forcing or pushing. The importance of patience, resilience and self-compassion throughout the recovery process are underlined. After the session participants are given access to a guided somatic future-focussed audio track to use in their own time.

**Session 8**

This session was an opportunity for participants to meet each other and the course lead in smaller informal groups of 5 - 6, to discuss challenges or issues with regard to their illness, applying the course content to their daily lives, using the various tools and strategies that were learned, and to share successes and strategies that had been effective for them so far. Following this session participants were provided with a booklet giving an overview and recap of the various tools covered in the course as they related to fatigue, post-exertional malaise, and chronic pain, and providing some examples and context recapping on how they can be implemented.

## Reported use of Intervention resources

Four questions at the end of the survey at T2 invited participants to indicate the extent to which they used the various resources provided on the course; i) Guided audio tracks (developed by lead author, including e.g. somatic tracking, somatic awareness, emotional awareness, imaginal reengagement), ii) Podcast recommendations (links to podcasts with experts in neural circuit disorders, and with people who have recovered from various illnesses such as long Covid and ME/CFS, using techniques/approaches similar to that covered in the TLC programme), iii) Handouts or documents developed by the course leader specifically for the TLC programme (including e.g. example of strategies for gradual reintegration of daily activities, strategies for working with fatigue, pain and PEM, and how to apply pacing when reintegrating activities), iv) Handouts or documents from other sources provided by the programme lead (including, for example, Schubiners (Schubiner, 2022) FIT self-diagnosis criteria sheet, and Alan Gordon’s Wiki Blog on neural circuit pain).

Responses varied by participant, and all participants indicated that they ‘frequently’ or ‘often’ used at least one resource type. As shown in Table S2 below, the Guided Audio tracks were most frequently used (with no participant citing ‘seldom/never’ for their usage).

**Table S2 Reported use of intervention resources at Post test (T2) (n = 10)**

|  | **Guided audio tracks** | **Podcast recommendations** | **Handouts/ documents developed by course leader** | **Handouts/ documents provided from other sources** |
| --- | --- | --- | --- | --- |
| **Frequently** | 4 | 3 | 2 | 1 |
| **Sometimes** | 6 | 4 | 5 | 5 |
| **Seldom/never** | 0 | 3 | 3 | 4 |

##

## Tidier Checklist

**
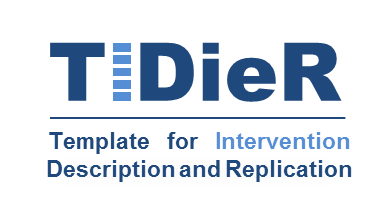
The TIDieR (Template for Intervention Description and Replication) Checklist*:**

Information to include when describing an intervention and the location of the information

| **Item number** | **Item** | **Where located **** | |
| --- | --- | --- | --- |
|  |  | Primary  paper | Other |
|  | **BRIEF NAME** |  |  |
| **1.** | Transforming Long COVID (TLC) programme – a neuroscience-informed group coaching and self-management intervention. | 1 |  |
|  | **WHY** |  |  |
| **2.** | The programme is grounded in theories of autonomic dysregulation, neural circuit disorders, and predictive processing, aiming to reduce symptom persistence through education, cognitive-emotional regulation, and self-management strategies. | 4, 6 | Supplementary  materials |
|  | **WHAT** |  |  |
| **3.** | Live online educational sessions, pre-recorded video sessions, guided audio tracks (somatic tracking, emotional awareness, visualisation), handouts, expressive writing guidelines, podcasts, and online shared drive resources. | 6 | Supplementary  materials |
| **4.** | Weekly online sessions combining neuroscience education, guided practices, group discussion, self-reflection, and home practice using video, audio and printed materials. | 6 | Supplementary  materials |
|  | **WHO PROVIDED** |  |  |
| **5.** | Delivered by the lead author, an academic with expertise in education, health literacy and physical activity, and a certified mind–body coach, meditation teacher, and chronic pain practitioner. | 5 | Supplementary  materials |
|  | **HOW** |  |  |
| **6.** | Delivered online via Zoom in group format, with supplementary self-paced materials accessed via shared drive. | 6 | Supplementary  materials |
|  | **WHERE** |  |  |
| **7.** | Online delivery using videoconferencing (Zoom) and cloud-based resource storage. | 6 | Supplementary  materials |
|  | **WHEN and HOW MUCH** |  |  |
| **8.** | Eight-week programme (October–December 2024): seven 90-minute content sessions and one 50–60 minute Q&A session, with weekly access to recordings. | 6 | Supplementary  materials |
|  | **TAILORING** |  |  |
| **9.** | Participants were encouraged to engage at their own pace, choose which tools to prioritise, and adapt practices to individual capacity and symptoms. | 6 | Supplementary  materials |
|  | **MODIFICATIONS** |  |  |
| **10.^ǂ^** | No major modifications were made during delivery; minor adaptations were made in response to participant needs (e.g., flexible attendance, camera use). | 6 | Supplementary  materials |
|  | **HOW WELL** |  |  |
| **11.** | Attendance was monitored, sessions followed a standardised structure, and all content was delivered by the same facilitator to maintain fidelity. | 8 | Supplementary  materials |
| **12.^ǂ^** | High adherence was observed, with 82% of participants attending at least six of seven sessions; all planned sessions were delivered. | 8 | Supplementary  materials |
